# Plasma *EGFR* T790M ctDNA status is associated with clinical outcome in advanced NSCLC patients with acquired EGFR-TKI resistance

**DOI:** 10.1038/srep20913

**Published:** 2016-02-12

**Authors:** D. Zheng, X. Ye, M. Z. Zhang, Y. Sun, J. Y. Wang, J. Ni, H. P. Zhang, L. Zhang, J. Luo, J. Zhang, L. Tang, B. Su, G. Chen, G. Zhu, Y. Gu, J. F. Xu

**Affiliations:** 1 Shanghai Pulmonary Hospital, Tongji University Medical School, Shanghai, China; 2 Asia & Emerging Markets Innovative Medicine, AstraZeneca R&D, Shanghai, China; 3 Research and Development Information, AstraZeneca, Shanghai, China

## Abstract

*EGFR* T790M mutation occurs in half of non-small cell lung cancer (NSCLC) patients with acquired EGFR-TKI (TKI) resistance, based on tumor re-biopsies using an invasive clinical procedure. Here, we dynamically monitored T790M mutation in circulating tumor DNA (ctDNA) using serial plasma samples from NSCLC patients receiving TKI through Droplet Digital PCR (ddPCR) method and the associations between overall survival (OS) starting from initial TKI treatment and the T790M ctDNA status detected in plasma were analyzed. Among 318 patients, 117 who acquired TKI resistance were eligible for the analysis. T790M ctDNA was detected in the plasma of 55/117 (47%) patients. Almost half of the T790M ctDNA positive patients were identified at a median time of 2.2 months prior to clinically progressive disease (PD). Furthermore, within the patients receiving TKI treatment at 2^nd^ line or later, the T790M ctDNA positive group had significantly shorter OS than the negative group (median OS: 26.9 months versus NA, P = 0.0489). Our study demonstrates the feasibility of monitoring *EGFR* mutation dynamics in serial plasma samples from NSCLC patients receiving TKI therapy. T790M ctDNA can be detected in plasma before and after PD as a poor prognostic factor.

The identification of *EGFR* activating mutations and the application of personalized first-line TKI therapy to NSCLC patients has led to a doubling of progression-free survival (PFS)^1^,^2^,^3^,^4^,^5^ and a lengthening of overall survival (OS) to more than 2 years, neither of which would have been achievable during the era of chemotherapy^6^,^7^. However, all patients with *EGFR*-mutant NSCLC ultimately develop PD while receiving TKI treatment. *EGFR* T790M mutation accounts for half of all reported resistance cases^8^,^9^,^10^, with other mechanisms including amplification of *MET*^11^,^12^, mutation of *PIK3CA*^13^ and *BRAF*^14^, transformation to small-cell lung cancer^13^,^15^ and epithelial-to-mesenchymal transition^13^. Therapeutic strategies have been developed based on these resistance mechanisms, emphasizing the importance of molecular characterization of post-TKI progression tumor samples. However, obtaining serial tumor re-biopsies following PD is challenging in clinical practice due to the invasiveness of the procedure, and also carries risk due to the limitations of the biopsy to reflect tumor heterogeneity and the evolution of genetic modifications^16^,^17^,^18^,^19^.

Several studies have demonstrated the potential for plasma-derived ctDNA to better represent the patient’s tumor genome, with mixtures of variants identified as originating from multiple tumor lesions including primary and metastatic sites^20^,^21^. Moreover, mutation detection in plasma has shown promise in terms of accessibility, convenience and practicality compared with analysis of isolated circulating tumor cells^22^,^23^. Detection rates of T790M ctDNA in plasma from NSCLC patients with acquired TKI resistance ranged from 30% to 50% using qualitative PCR-based assays^24^,^25^,^26^,^27^, but the prognostic role of T790M ctDNA status in salvage therapies for acquired TKI resistance has not been evaluated. Using a droplet digital PCR (ddPCR) method, detectable quantitative changes in T790M ctDNA within weeks prior to PD on TKI was recently reported in 9 NSCLC patients^28^. The clinical significance of this data was not addressed however, as sample data beyond TKI failure was unavailable.

Herein, using ddPCR assay we evaluated the dynamic changes in *EGFR* mutation status in advanced NSCLC patients’ plasma, up to and beyond TKI failure. Furthermore, our study shows for the first time, an association between T790M ctDNA emergence in plasma and the patients’ OS since the initial TKI therapy.

## Results

### Patient characteristics and plasma sample collection

**Figure 1:**
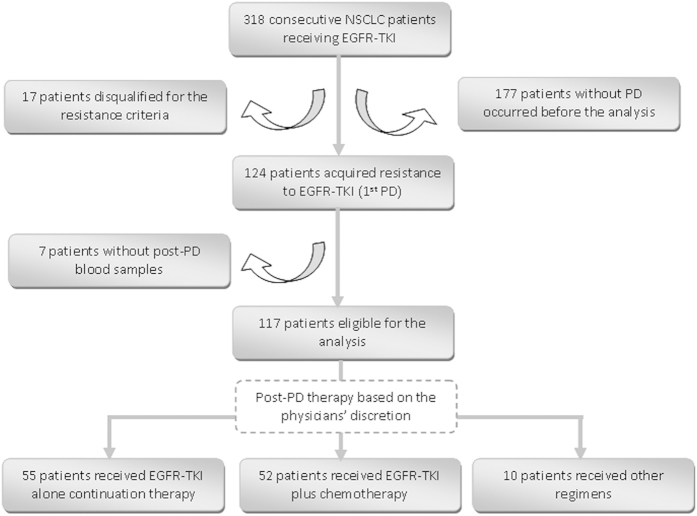
Patient enrollment flow chart. NSCLC, non-small cell lung cancer; EGFR-TKI, epidermal growth factor receptor-tyrosine kinase inhibitor; PD, progressive disease.

318 patients with advanced or recurrent NSCLC who were receiving TKI or intended to be given TKI since May 2013 in Shanghai pulmonary hospital were enrolled consecutively to our study (Fig. 1). These patients began TKI therapy between Nov 2006 and Sept 2013, and developed PD from Sept 2011 to Apr 2014. By the cut-off date, 124 patients had acquired TKI resistance. 7 patients were excluded for further analysis due to lack of post-PD plasma samples. At the time of enrollment, 41 patients were receiving continued TKI treatment beyond failure, while the other 76 patients were either TKI-naïve or still responding to initial TKI treatment. After failing initial TKI treatment, all 117 patients received either TKI alone continuation therapy (TKI alone, n = 55), TKI plus chemotherapy (Combo, n = 52), or other therapies (Others, n = 10). Due to a lack of standard of care options for TKI-relapsed patients, salvage treatments were selected at the physicians’ discretion. Patients who failed first line TKI therapy received platinum-based doublet regimens, whilst those who failed second or later-line TKI therapy received single agent chemotherapy. The median follow-up time was 492 days (range, 81 to 2661).

Serial blood samplings were taken every 2 months during outpatient evaluation follow-up, however blood samples were not available at every visit for all patients. A total of 391 plasma samples from 117 patients were collected for *EGFR* mutation testing. The distribution of blood samplings from different time periods against 1^st^ PD was summarized in Supplemental Figure S1.

### Validation of ddPCR assays for detecting *EGFR* mutations

Highly sensitive ddPCR assays for testing *EGFR* mutations including 19Dels, L858R and T790M were developed to an analytical sensitivity of 0.04% (Supplemental Figure S2).

As we recently reported^29^, the detection sensitivity and specificity of plasma *EGFR* mutations using ddPCR assay, as compared to the paired tumor tissues, were 81.82% and 98.44% for 19Dels, and 80.00% and 95.77% for L858R, respectively. Here, we evaluated the detection sensitivity and specificity of plasma T790M mutation using the newly developed ddPCR assay, by comparison with the T790M status in the paired tumor re-biopsy or PE samples (referred to as “tumor tissue”) as standard. The T790M status in 25 pairs of tumor tissue and plasma from EGFR-TKI relapsed NSCLC patients is summarized in Table 1. Among these patients, 13 were positive for T790M in both tumor tissue and plasma, and 9 were negative in both. The other 3 patients were positive for T790M in tumor tissue, but negative in plasma. Together, the overall concordance rate of T790M testing between the paired tumor tissues and plasma was 88.00% (22/25) (Kappa = 0.757, 95%CI: 0.4996–1.0). The sensitivity and specificity of plasma T790M testing by ddPCR assay were 81.25% (13/16) (95%CI: 54.35%–96.00%) and 100.00% (9/9) (95%CI: 66.37%–100%), respectively.

### Dynamic detection of *EGFR* mutations in patients’ plasma

Patients were characterized as positive for *EGFR* 19Dels, L858R and/or T790M when mutant ctDNA was detected in plasma at any timepoint during the study. Overall, T790M ctDNA mutation in plasma was detected in 55 (47%) of the 117 patients. Based on plasma T790M status, all 117 patients were categorized into either T790M positive or negative groups. There was no significant difference in clinical parameters between these two groups, including age, sex, histology, TNM staging, smoking history, line of TKI treatment and post-PD therapies (Table 2).

**Figure 2:**
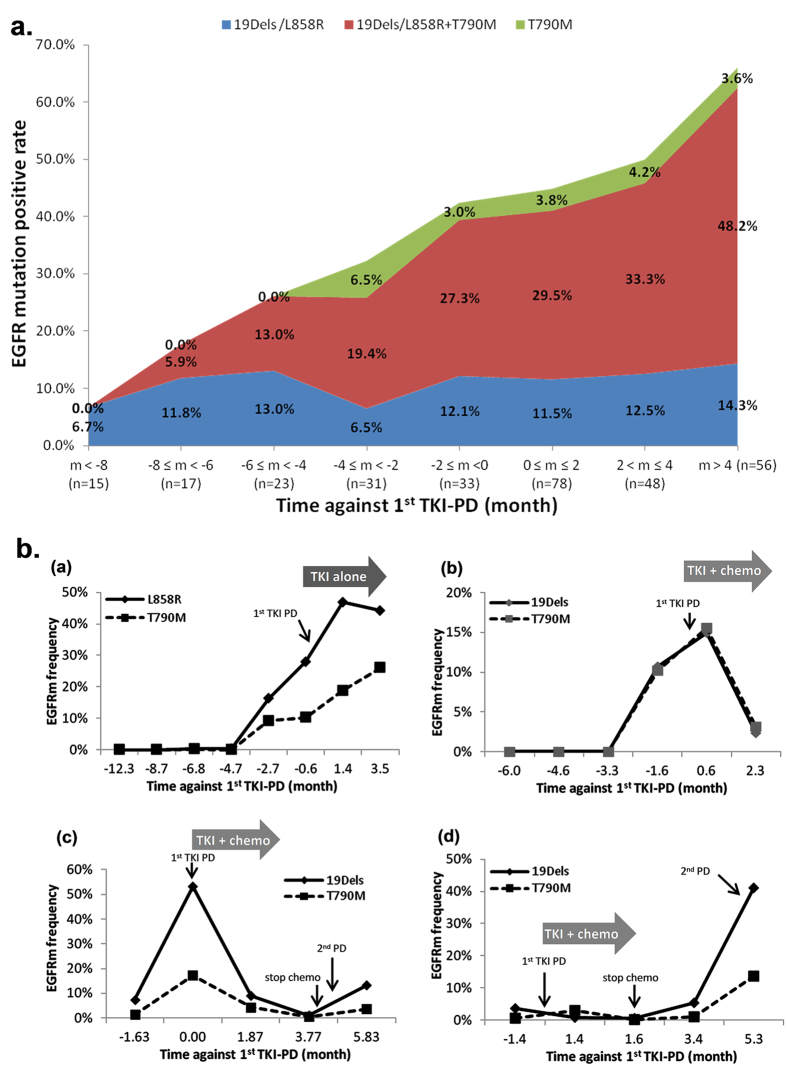
Dynamic detection of *EGFR* mutant ctDNA in plasma by ddPCR. (**a**) Dynamic monitoring of *EGFR* mutant ctDNA status in plasma from 117 patients before and after EGFR-TKI progression. Patients were grouped every 2 months against 1^st^ PD. Y-axis refers to the percentage of patients positive for the *EGFR* mutation status, 19Dels or L858R alone (blue), 19Dels or L858R plus T790M (red), and T790M alone (green). “n” refers to the number of patients. (**b**) Monitoring of response and resistance to clinical treatments by serial measurement of plasma *EGFR* ctDNA. Emergence of *EGFR* mutant ctDNA was detected in plasma prior to clinical PD and the mutation incidence increased along with disease progression (**a**,**b**) Decreases in *EGFR* mutant ctDNA in response to post-PD TKI plus chemotherapy was observed in (**b**–**d**) Re-emergence and accumulation of *EGFR* mutant ctDNA were observed after withdrawal of chemotherapy.

As the duration of TKI treatment and the frequencies of blood sampling varied among patients, the *EGFR* mutation results from the plasma samples were grouped every 2 months against the 1^st^ PD of each individual patient for prevalence analysis (Fig. 2a). “n” refers to the number of patients who had blood sampling collected within each time period. For the time periods with more than 1 plasma sampling, such as m<-8 and m>4, individual patient was characterized as positive when at least one plasma sample showed positive detection of *EGFR* mutation. Three subgroups of *EGFR* mutation positive patients were identified, including 19Dels or L858R alone, 19Dels or L858R plus T790M, and T790M alone. The patients with *EGFR* 19Dels or L858R plus T790M accounted for the majority of the *EGFR* mutant patients. The prevalence of this subgroup genotype increased gradually with disease progression while still on continuous TKI treatment, from 5.9% at 6-8 months before PD to 48.2% at 4 months after PD and beyond. In addition, *EGFR* 19Dels or L858R alone was found in 6.5% to 14.3% of the patients in plasma throughout the study. T790M alone was also detected in 3–6.5% of the patients. Finally, the incidence of patients negative for any *EGFR* mutation at 4 months beyond PD was 33.9% (19/56). In summary, our results showed that the majority of T790M positive patients also simultaneously carried 19Dels or L858R in plasma throughout the course of their disease.

### Monitoring of plasma T790M before 1^st^ PD upon initial TKI treatment

Among the 76 patients who had pre-PD plasma samples available, 35 (46.0%) were positive for T790M, from which 16 were identified based on plasma samples collected prior to PD. This suggests that almost half (16/35, 45.7%) of the patients who acquired T790M-mediated TKI resistance could be identified earlier using plasma ctDNA analysis before clinical PD. In summary, the median time of early detectable T790M in plasma was 2.2 months before clinical PD (range: 0.8 to 6.8 months) in these patients.

### Monitoring of plasma T790M after 1^st^ PD upon initial TKI treatment

It remains unclear whether T790M ctDNA could be detected persistently in the serial post-PD plasma samples. In this study, 35 patients positive for T790M had at least 2 post-PD plasma samples. 14 (14/35, 40%) had consistently detectable T790M ctDNA in all available post-PD plasma samples. Variation of T790M status in the serial post-PD plasma samples was found in the other 21 patients, and was associated with either late T790M ctDNA emergence (13/21) and/or the elimination of T790M ctDNA by the post-PD therapies (examples shown in Fig. 2b).

One of the advantages of ddPCR is its ability to quantify ctDNA in plasma. We evaluated whether changes in *EGFR* ctDNA level in the serial plasma samples was associated with the patient’s disease course, including treatment response. In patient 1 who had been receiving TKI mono-therapy before and after PD (Fig. 2b-(a)), the emergence of L858R and T790M ctDNA was observed in the plasma at 2.7 months before clinical PD, and the proportion of both mutants increased along with disease progression. In patient 2 (Fig. 2b-(b)), 19Dels and T790M ctDNA were detected at the same level in plasma starting at 3.3 months before PD, increasing to peak levels around the time of PD, and subsequently dropping following initiation of post-PD TKI plus chemotherapy treatment. In patients 3 and 4 (Fig. 2b–d)), 19Dels and T790M ctDNA were detected around the time of PD and then decreased to an undetectable level in response to post-PD TKI plus chemotherapy treatment. After chemotherapy withdrawal, 19Dels and T790M ctDNA re-emerged and increased rapidly in the plasma.

### Association of plasma T790M status with patient survival

**Figure 3:**
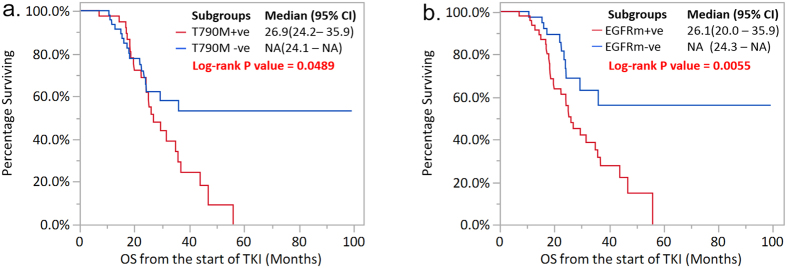
Overall survival in the patients receiving TKI treatment at 2^nd^ line or later according to (**a**) T790M status in plasma; (**b**) post-PD *EGFR* mutation status in plasma. All the 88 patients in the 2^nd^ line or later TKI treatment subgroup had evaluable T790M ctDNA status (T790M+ve, n = 40; T790M–ve, n = 48), and 87 patients had evaluable post-PD *EGFR* mutation status (*EGFRm*+ve, n = 49; *EGFRm*–ve, n = 38). *P < 0.05.

**Figure 4:**
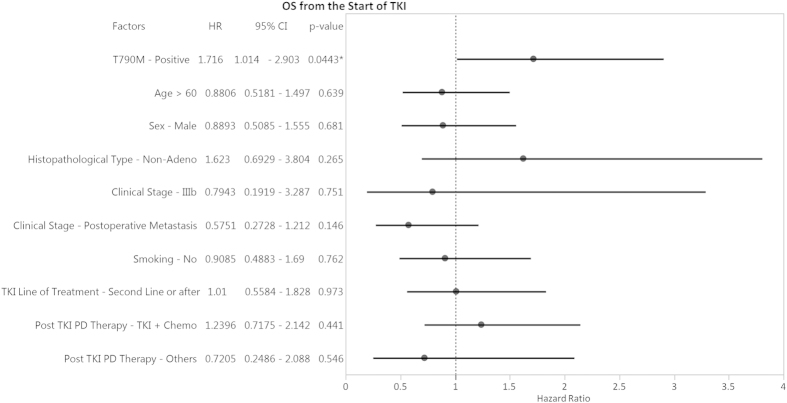
Forest plot of hazard ratios (HR) for overall survival according to patient’s clinical characteristics, plasma T790M status and post-PD therapy. *P < 0.05; ■ 0.05 <P < 0.1. This figure shows the result of univariate analysis only. Multivariate analysis was carried out with the full model followed by a backward stepwise selection process by AIC. AIC value = 455.36. A model containing only plasma T790M status was selected to be the best model. Hence, no multivariate analysis result is shown here.

OS was assessed in all 117 patients based on their plasma T790M status and TKI treatments. For patients receiving TKI treatment at 2^nd^ line or later, those with detectable T790M ctDNA had worse survival with a median OS of 26.9 months, compared to those patients without T790M ctDNA with a median OS that had not yet been reached (majority of the T790M negative patients are censored alive) (P = 0.0489, Fig. 3a). A similar trend was also observed in those patients receiving 1^st^ line TKI treatment, although statistical significance was not reached due to the small size of this group (Supplemental Figure S3a). Consistently, univariate and multivariate Cox proportional hazard model analyses identified plasma T790M positivity as an independent prognostic factor for a worse OS in these patients (HR = 1.716, 95% CI: 1.014 – 2.903, P = 0.0443, Fig. 4).

Next we investigated the prognostic role of the patient’s plasma *EGFR* mutation status after PD, regardless of the mutation type. Interestingly, plasma *EGFR* mutation positivity (*EGFRm*+) after PD was also found to be a negative prognostic factor for those patients receiving TKI treatment at 2^nd^ line or later. For the patients positive or negative for any detectable *EGFR* mutation in their post-PD plasma, the median OS was 26.1 months compared to ‘not yet reached’, respectively (P = 0.0055, Fig. 3b) - (the majority of the patients in this group were censored alive). Again, a similar trend was also observed for patients receiving 1^st^ line TKI treatment (Supplemental Figure S3b).

## Discussion

With the rapid advent of genotype directed therapies aimed at overcoming acquired TKI resistance, it is becoming imperative to accurately and quickly identify distinct molecular resistance mechanisms within individual patients. The secondary *EGFR* T790M mutation has been discovered in tumor re-biopsies from around half of all NSCLC patients with acquired resistance to TKI therapy^8^,^9^,^10^,^30^,^31^,^32^. Consistent with this, in our study of patient plasma samples, we detected T790M ctDNA in 47% (55/117) of patients with PD on initial TKI therapy, suggesting that a highly sensitive ddPCR method could provide an alternative approach to detecting plasma T790M status, especially in situations where tumor re-biopsy may be challenging.

Our study demonstrated that half of all patients with plasma T790M ctDNA were identified at a median time of 2.2 months prior to clinical PD upon initial TKI treatment, a finding which is consistent with the recent report by Oxnard’s group^28^. These findings raise a key question to be addressed in future studies namely, ‘should targeted therapy against T790M be administered upon T790M ctDNA emergence in plasma, even without clinical PD according to the RECIST criteria?’.

The plasma T790M positivity rate increased progressively from 6 months prior to PD to 4 months beyond PD. In parallel with the progressively increasing T790M ctDNA detected in plasma, the *EGFR* sensitive mutations (19Dels or L858R) also increased from 6.7% to 62.5%. This supports the notion that T790M is acquired during the evolution of cancer cells under selection due to long-term TKI exposure and mostly coexists with 19Dels or L858R mutation in the same cells, as reported by the previous studies^10^,^11^,^12^,^33^,^34^. This underscores the likely growth advantage of double mutant tumor cells under sustained TKI selective pressure^34^,^35^,^36^.

Of particular clinical interest is the hypothesis that a higher prevalence of T790M positive cells can contribute to tumor outgrowth and therefore clinical outcome, including TKI resistance. In an *in vitro* study, Chmielecki *et al.* investigated the effect of T790M positive clone percentage on TKI sensitivity^35^. The authors found that with TKI treatment, the mixed cell population displayed accelerated proliferation only when the T790M positive cell proportion reached >10% of the total cell population, and that sensitivity to TKI was reduced only when the T790M positive cell proportion became >25%. Moreover, Youngjoo Lee *et al.* demonstrated that patients with a high T790M tumor mutation frequency had significantly worse clinical outcomes in response to TKI therapy^36^. In the present study, we found that positive plasma T790M ctDNA detection in patients receiving TKI was a poor prognosis factor. This indicates that detection of T790M ctDNA in plasma is likely a consequence of T790M positive tumor cells exceeding the required threshold for enabling tumor outgrowth, and may also reflect the increase of tumor burden and metastasis. Likewise, from the exploratory biomarker analysis of the FAST-ACT II study, Mok TS *et al.* recently demonstrated that *EGFR* mutations detected in plasma from patients who received 3 cycles of treatment was predictive for worse PFS and OS^37^, and was correlated with change in tumor burden or increased metastases. In our study, T790M mostly coexisted with 19Dels or L858R, and the positivity of *EGFR* mutant ctDNA in plasma was also a poor prognostic factor for patients with acquired TKI resistance. One could suggest that a patient’s plasma T790M status upon TKI failure, which mostly coexists with an *EGFR* activating mutation, is associated with increased tumor burden and/or metastasis, thereby leading to poor survival.

On the other hand, several retrospective studies of tumor re-biopsies have demonstrated that NSCLC patients who acquired TKI resistance due to T790M had longer survival than patients without T790M^30^,^31^,^32^. This discrepancy suggests that plasma T790M status should not simply be considered as a surrogate of tumor mutation status in patients. This can likely be explained by our limited understanding of ctDNA biology in patients, including the timings and mechanisms of release of T790M ctDNA into plasma from primary and metastatic tumor lesions. For example, the time interval between TKI progression and re-biopsy sampling might cause the different T790M status from the plasma. Moreover, considering the temporal and spatial heterogeneity of tumor tissue genetic profile, a discrepancy of T790M status from the plasma might exist. Furthermore, considering the feasibility and accessibility of tissue re-biopsy versus blood sampling, the studied patient populations and the mutation detection approaches used in our study and others were likely different.

In this study, one perceived challenge was the lack of real-time matched tumor tissues available to explore the timing of emergence and quantification of T790M mutation as compared to plasma. However, considering the reported intra-patient heterogeneity of T790M in multifocal synchronous or metachronous tumors^38^,^39^, we believe that the comprehensive profiling of TKI-resistance mechanisms could be better performed using plasma ctDNA analysis of serial blood samples compared to single tumor re-biopsies.

In summary, we have demonstrated the feasibility of monitoring *EGFR* mutation dynamics in serial plasma samples from NSCLC patients receiving TKI therapy in real-world clinical practice. We have also shown that detection of plasma T790M ctDNA in patients with acquired TKI-resistance is a poor prognostic factor, especially in those patients receiving TKI treatment at 2^nd^ line or later. Our results highlight the clinical utility of plasma T790M ctDNA detection in guiding TKI therapies for *EGFR* mutant NSCLC patients, and support further validation of this approach in future prospective interventional multi-center clinical studies.

## Materials and Methods

### Patients and samples

Patients with advanced or recurrent NSCLC who had been receiving (or were about to receive) TKI treatment from May 2013 at the Shanghai Pulmonary Hospital were enrolled consecutively. Following informed consent, patient blood samples were drawn every 2 months during TKI treatment and beyond PD. Pleural effusion (PE) or tumor re-biopsy samples were collected when available from the patients with acquired TKI resistance. Patients who met the following criteria for acquired resistance to TKI were included for analysis^40^: patients showing response or durable stable disease (>6 months) on TKI followed by progression whilst receiving TKI; or patients with documented *EGFR* activating mutation who developed PD during TKI treatment. Patients received TKI alone continuation therapy, TKI plus chemotherapy or others upon initial TKI-PD (referred to as ‘1^st^ PD’ hereafter) at the physicians’ discretion. Treatment effectiveness was assessed every 2 months according to RECIST 1.1. *EGFR* mutation testing in plasma and imaging studies (including Chest CT, brain MRI, bone scan and abdominal ultrasound) were performed by two independent research teams who were blinded from each other until survival outcome was analyzed. Survival status was confirmed by regular telephone follow-up every 3 months. The study was approved by Shanghai Pulmonary Hospital research ethics committee. In addition, the experiments in this study were conducted in accordance with approved guidelines and regulations.

### Plasma isolation and DNA extraction

Patient blood samples were collected in tubes containing trisodium citrate and centrifuged at 2500 g for 10 min at 4 °C within 2 hours of collection. The plasma supernatant was isolated and stored at −80 °C. PE samples, when available, were collected and centrifuged at 2500 g for 10 min at 4 °C within 2 hours of collection. Supernatant and cell pellets from PE were stored in two individual tubes at −80 °C. Cell free DNA from 1.5 ml plasma and PE supernatant samples was extracted with QIAamp Circulating Nucleic Acid kit (Qiagen, Hilden, Germany). DNA from PE cell pellets was extracted with a Puregene DNA extraction kit (Qiagen).

### *EGFR* mutation detection

Development of ddPCR assays for *EGFR* Exon19-Dels (19Dels) and L858R mutations has been described previously^29^. Details of the ddPCR assay for *EGFR* T790M mutation detection were described in supplemental method 1. The calculation method for plasma DNA input was provided in supplemental method 2. PCR reaction conditions and quantification of *EGFR* mutant ctDNA fraction were also described in supplemental method 2.

*EGFR* T790M status in tumor re-biopsy or PE samples was determined by using ARMS kit (AmoyDx, Xiamen, China)^29^ or the ddPCR assay above.

### Statistical analyses

The sensitivity and specificity of the plasma T790M test using ddPCR assay was determined by comparison of T790M status in the paired tumor re-biopsy or PE samples. Calculation details regarding consistency rates and confidence intervals for sensitivity and specificity were previously described^29^. Demographic and clinical characteristics based on the T790M ctDNA status were compared using the Fisher’s Exact Test. OS was calculated from initiation of TKI treatment to death for any reason, or the last follow up date (censored) and presented as median (95% confidence interval). Survival data were analyzed using the Kaplan-Meier method. OS of patients from different subgroups were compared using the log-rank test. The Cox proportional hazard model was applied to examine whether post-PD therapies, T790M status in plasma and patients’ characteristics were predictor(s) for OS. Significance was established when P values were less than 0.05. All tests were two-sided. Statistical analyses and data visualization were performed using R version 3.0.2 and JMP version 11.0.0 (SAS Institute Inc).

## Additional Information

**How to cite this article**: Zheng, D. *et al.* Plasma *EGFR* T790M ctDNA status is associated with clinical outcome in advanced NSCLC patients with acquired EGFR-TKI resistance. *Sci. Rep.* **6**, 20913; doi: 10.1038/srep20913 (2016).

## Supplementary Material

Supplementary Information

## Figures and Tables

**Table 1:** Comparison of *EGFR* T790M detected in plasma versus the status detected in paired tumor tissue.

Tumor tissue	Plasma		Total
	+	−	
+	13	3	16
−	0	9	9
Total	13	12	25

**Table 2:** Clinical characteristics and *EGFR* T790M ctDNA status in plasma of 117 patients.

	Total	T790M ctDNA in plasma		p-value
		T790M +ve	T790M −ve	
N	117	55	62	
Age				1
<=60	66	31	35	
>60	51	24	27	
Sex				0.1293
Female	71	29	42	
Male	46	26	20	
Histopathological type				0.1698
Adenocarcinoma	108	53	55	
Non-adeno	9	2	7	
Clinical stage				0.0677
Recurrent	21	6	15	
Stage IV	91	48	43	
Stage IIIb	5	1	4	
Smoking				0.3918
Yes	29	16	13	
No	88	39	49	
TKI line of treatment				0.6688
First line	29	15	14	
Second line or after	88	40	48	
Post TKI PD therapy				0.6467
TKI alone	55	24	31	
TKI + chemo	52	25	27	
Others	10	6	4	
