# Supplementary material for: Plasma EGFR T790M ctDNA status is associated with clinical outcome in advanced NSCLC patients with acquired EGFR-TKI resistance
Source: Sci Rep. 2016 Feb 12;6:20913. doi: 10.1038/srep20913 (PMC4751431; doi:10.1038/srep20913)
Supplement: Supplementary Information [file srep20913-s1.pdf]

# Plasma *EGFR* T790M ctDNA status is associated with clinical outcome in advanced NSCLC patients with acquired EGFR-TKI resistance

[1#†]D Zheng; [2#]X Ye; [3]MZ Zhang; [2]Y Sun; [1†]JY Wang; [1†]J Ni; [1†]HP Zhang; [1†]L Zhang; [1†]Jie Luo; [1‡]J Zhang; [1‡]L Tang; [1‡]B Su; [1ǂ]G Chen; [2]GS Zhu; [2*]YGu; [1*]JF Xu.

[1]Shanghai Pulmonary Hospital, Tongji University Medical School, Shanghai, China

[†] Department of Medical Oncology, Shanghai Pulmonary Hospital.

[‡] Central Laboratory, Shanghai Pulmonary Hospital.

[ǂ] Department of Pathology, Shanghai Pulmonary Hospital.

[2]Asia & Emerging Markets Innovative Medicine, AstraZeneca R&D, Shanghai, China

[3]Research and Development Information, AstraZeneca, Shanghai, China

***Corresponding authors**: xujianfang63@aliyun.com and yi.gu@astrazeneca.com

# D. Zheng and X. Ye contributed equally to this study.

SUPPLEMENTAL METHODS

## 1. Development of the ddPCR assays for testing *EGFR* 19Dels, L858R and T790M mutations

The development of ddPCR assays for *EGFR* Exon19-Dels (19Dels) and L858R has been described previously[30]. For the *EGFR* T790M ddPCR assay, the sequences of primers and probes are below and the design principle is shown in supplemental Figure 2A:

Forward primer: 5'- CCTCACCTCCACCGTGCA-3'

Reverse primer: 5'- AGGCAGCCGAAGGGCA-3'

Mutant probe: 5'-FAM-AGCTGCATGATGA-MGB-3'

Wild type probe: 5'-VIC-AGCTGCGTGATGA-MGB-3'

To evaluate the sensitivity of the T790M assay, DNA from NCI-H1975 cells (harboring T790M mutation) was serially diluted in human reference genomic DNA to achieve decreasing ratios (1:1 to1:10000) of T790M mutant allele versus wild type allele. The final 20 µL of TaqMan PCR reaction mixture was assembled with 1× ddPCR Master mixture (Catalog No. 1863010, Bio-Rad Laboratories), 900 nM of each primer, 450 nM of each probe, and 50 ng DNA templates. Each assembled ddPCR reaction mixture was subjected to droplet generation followed by PCR reaction. Thermal cycling conditions were as follows: 10-min incubation at 95°C followed by 45 cycles of 95°C for 15 sec, 60°C for 1 min, and then 4°C hold. Droplet fluorescence was collected in the droplet reader. Analysis of ddPCR data for allele calling was performed with QuantaSoft software version 1.3.2.0 (Bio-Rad). Human reference genomic DNA (Catalog No. G1471, Promega) was routinely included as a negative control and used to determine the cut-off for allele calling.

## 2. *EGFR* ctDNA detection in plasma using the ddPCR assay

The reaction mix was prepared as described above. Plasma cell free DNA, 7.3 uL from each, was loaded into the reaction mix. The number of positive droplets and

sample input follows the Poisson distribution. Calculation of plasma sample DNA input per reaction (*I*, copies per reaction) was calculated with the equation:

$$I\ (copies/reaction) = (-\ln(1-\mathrm{p})/\mathrm{V}) * 1000 * 20$$

p: fraction of positive droplets; V: volume of each droplet (0.91 nl)

For 19Dels assay, *I* equals to the copies of *EGFR*-Exon2 DNA template (VIC signal). For L858R and T790M assays, *I* equals to the total copies of *EGFR* mutant and wild type DNA templates (FAM and VIC signal). The PCR thermal profile was the same as mentioned in the section above. Four human reference genomic DNA samples were used as negative controls. Two positive controls with 1:2500 ratio of mutant allele to wild type allele and two non template controls (NTC) were always included in each run. The samples were called positive for target mutations when they contained at least 2 droplets in the positive area of FAM signal. The fraction of *EGFR* L858R or T790M mutant (F1) was calculated as below:

$$F1 = \frac{I\ (\mathrm{FAM})}{I\ (\mathrm{FAM}) + I\ (\mathrm{VIC})}$$

The fraction of *EGFR* 19Dels mutant (F2) was calculated as below:

$$\mathrm{F2} = \frac{I\ (\mathrm{FAM})}{I\ (\mathrm{VIC})}$$

SUPPLEMENTAL FIGURES

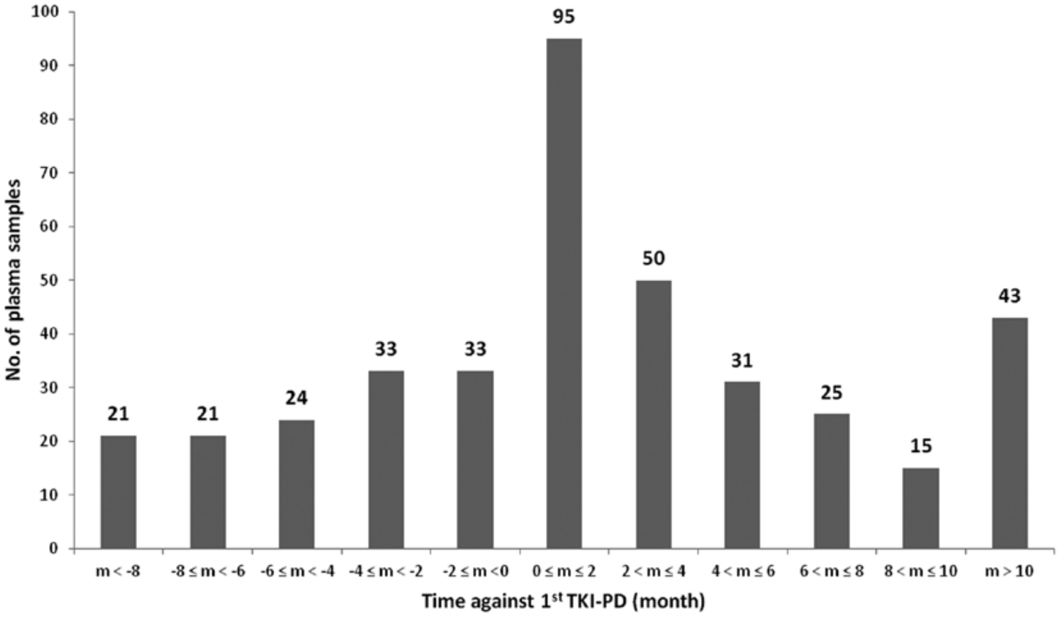


**Supplemental Figure S1**. Distribution of patient's plasma samples collected during different time period against 1st PD upon initial TKI treatment.

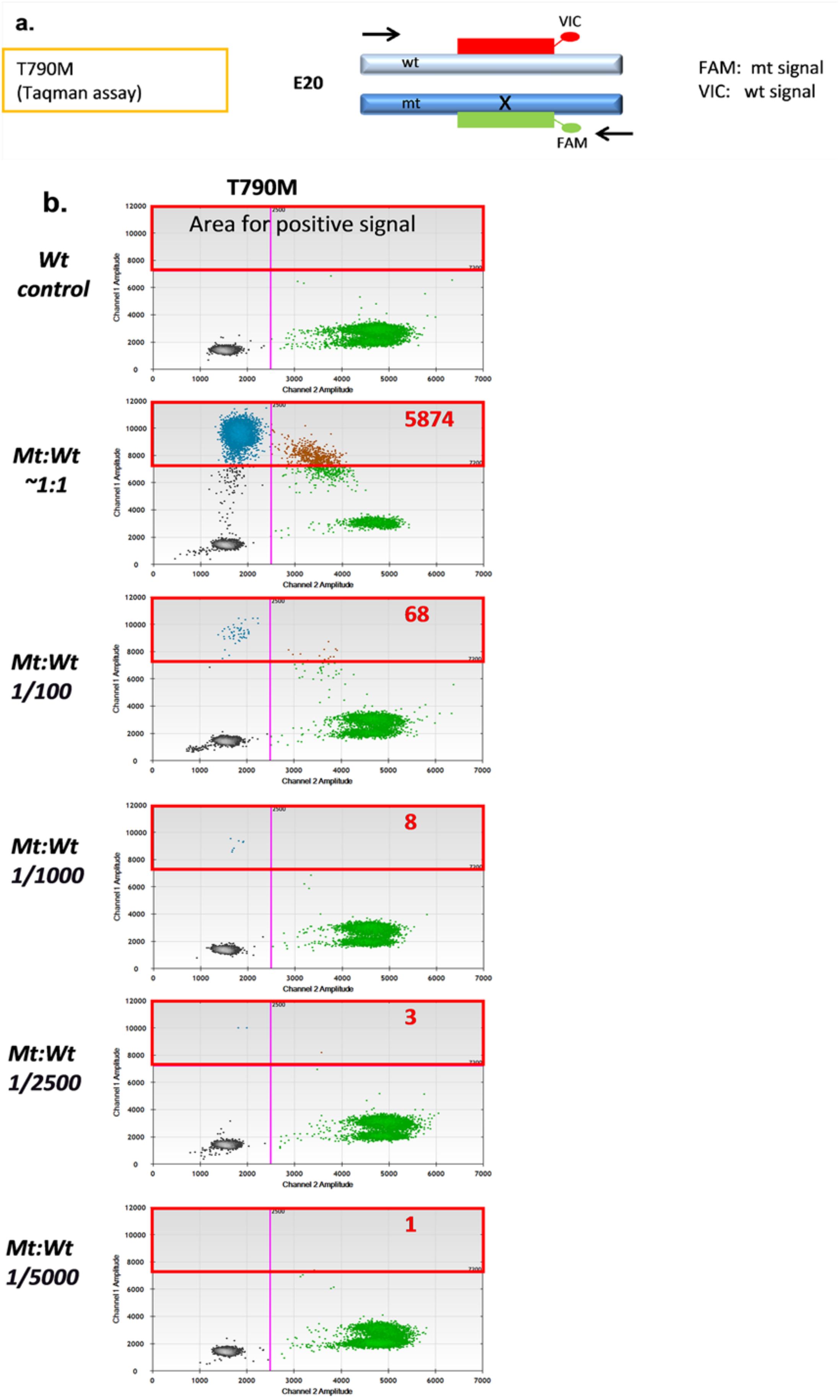
a.
T790M
(Taqman assay)
E20
VIC
wt
mt
X
FAM
FAM: mt signal
VIC: wt signal
b.
T790M
Area for positive signal
Wt
control
Mt:Wt
~1:1
5874
Mt:Wt
1/100
68
Mt:Wt
1/1000
8
Mt:Wt
1/2500
3
Mt:Wt
1/5000
1
Channel 1 Amplitude
Channel 2 Amplitude

**Supplemental Figure S2.** Design and evaluation of the ddPCR assay for *EGFR* T790M mutation detection. a. Design principle of the T790M ddPCR assay. FAM- and VIC-labeled MGB probes were designed to target mutant and wild type DNA templates of T790M mutation region of *EGFR* exon 20, respectively. b. Sensitivity evaluation of the T790M ddPCR assay. The T790M ddPCR assay was able to stably detect positive droplets with up to 1/2500 sensitivity. mt, mutant; wt, wild type.

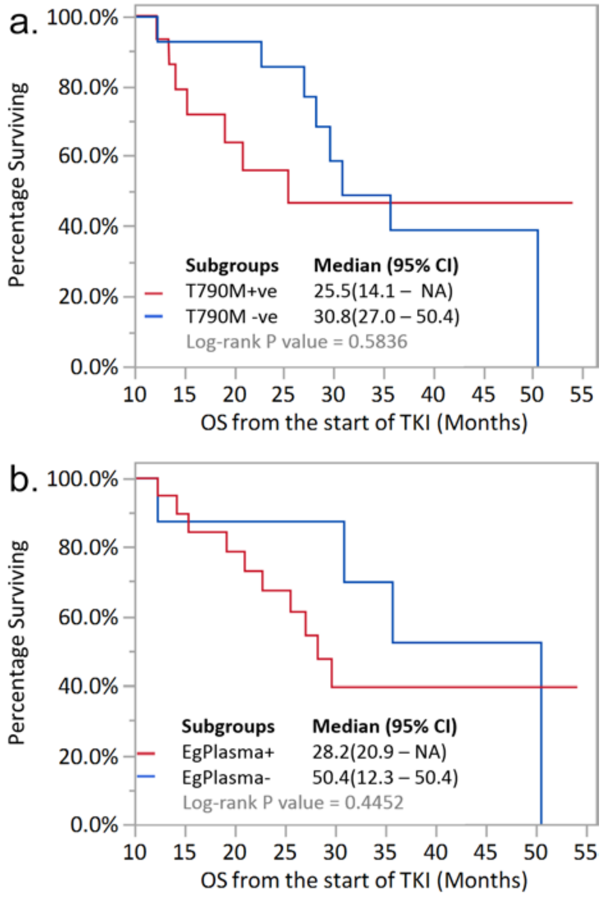


**Supplemental Figure S3.** Overall survival in the 1st line TKI treatment subgroup according to (a) T790M status in plasma and (b) post-PD *EGFR* mutation status in plasma. All 29 patients in the 1st line TKI treatment subgroup had evaluable T790M

ctDNA status (T790M+ve, n=15; T790M-ve, n=14), and the 28 patients had evaluable post-PD *EGFR* mutation status (*EgPlasma*+, n=20; *EgPlasma*+, n=8). * $P < 0.05$.
